# The interaction of BDNF with estrogen in the development of hypertension and obesity, particularly during menopause

**DOI:** 10.3389/fendo.2024.1384159

**Published:** 2024-11-25

**Authors:** Zhongming Zhang, Ziyi He, Jing Pan, Minghui Yuan, Yini Lang, Xiaomeng Wei, Chaoyun Zhang

**Affiliations:** ^1^ Zhang Zhongjing College of Chinese Medicine, Henan Key Laboratory of Zhang Zhongjing’s Formulas for Immunoregulation, Nanyang Institute of Technology, Nanyang, Henan, China; ^2^ School of Medicine, Zhengzhou University of Industrial Technology, Xinzheng, Henan, China; ^3^ The First Clinical College, Shandong University of Traditional Chinese Medicine, Jinan, Shandong, China

**Keywords:** BDNF, estrogens, hypertension, obesity, interplay

## Abstract

The expression of BDNF in both neuronal and non-neuronal cells is influenced by various stimuli, including prenatal developmental factors and postnatal conditions such as estrogens, dietary habits, and lifestyle factors like obesity, blood pressure, and aging. Central BDNF plays a crucial role in modulating how target tissues respond to these stimuli, influencing the pathogenesis of hypertension, mitigating obesity, and protecting neurons from aging. Thus, BDNF serves as a dynamic mediator of environmental influences, reflecting an individual's unique history of exposure. Estrogens, on the other hand, regulate various processes to maintain overall physiological well-being. Through nuclear estrogen receptors (ERα, ERβ) and the membrane estrogen receptor (GPER1), estrogens modulate transcriptional processes and signaling events that regulate the expression of target genes, such as ERα, components of the renin-angiotensin system (RAS), and hormone-sensitive lipase. Estrogens are instrumental in maintaining the set point for blood pressure and energy balance. BDNF and estrogens work cooperatively to prevent obesity by favoring lipolysis, and counteractively regulate blood pressure to adapt to the environment. Estrogen deficiency leads to menopause in women with low central BDNF level. This review delves into the complex mechanisms involving BDNF and estrogen, especially in the context of hypertension and obesity, particularly among postmenopausal women. The insights gained aim to inform the development of comprehensive therapeutic strategies for these prevalent syndromes affecting approximately 68% of adults.

## 1 Introduction

Brain-derived neurotrophic factor (BDNF) is highly expressed in the brain and moderately expressed in the heart, lungs, and kidneys. Numerous investigations confirm that BDNF has extensive roles by binding to its specific receptor, tropomyosin-related kinase receptor B (TrkB). This binding leads to the autophosphorylation of tyrosine residues in TrkB, initiating multiple signaling cascades, including rat sarcoma (RAS)-mitogen-activated protein kinases (MAPK) pathway, the phosphatidylinositol 3-kinase (PI3K)-Akt pathway, and the phospholipase C (PLC)-Ca^2+^ pathway (1–3). The MAPK pathway promotes neuronal differentiation and growth, the PI3K-Akt pathway is essential for cell survival, and PLCγ activation leads to the production of inositol 1,4,5 trisphosphate (IP3) and diacylglycerol (DAG), which subsequently activate Ca^2+^/calmodulin-dependent protein kinases and protein kinase C (PKC) pathways respectively (4). Physiologically, BDNF is well-known for its essential role in various neuronal processes during prenatal development, growth, maintenance, and the plasticity of the nervous system. Moreover, it also exerts non-neuronal effects on normal physiology and has been implicated in the pathogenesis of obesity and hypertension (5).

Estrogens, primarily produced in the follicular granulosa cells in premenopausal women and the stromal cells of adipose tissue in postmenopausal women, exert their effects by binding to specific receptors—the nuclear estrogen receptors (ERα, ERβ) and the membrane estrogen receptor (GPER1). These receptors activate transcriptional processes either as coregulators or through signaling pathways involving G protein activation and the cross-activation of MAPK, PI3K-Akt, and PKC, ultimately regulating gene expression and/or enzyme activity (6–8). Genes regulated by estrogen, known as estrogen-responsive genes, include BDNF (9), renin (10), estrogen receptor α (ERα) (11), angiotensinogen (12), hormone-sensitive lipase (HSL), proadipogenic genes such as peroxisome proliferator-activated receptor γ (PPARγ), steroid receptor coactivator-1 (SRC-1), and CREB-binding protein (CBP) (13, 14), as well as adipogenic genes like fatty acid synthase (FASN) (15). Estrogens are vital for both reproductive and non-reproductive functions (16), significantly influencing sexually dimorphic traits and renin expression through ERα (10). Dysregulation or deficiency of estrogen, particularly in postmenopausal women, can lead to conditions such as hypertension and obesity, common symptoms associated with menopause (17, 18).

BDNF and estrogens demonstrate multifaceted interactions that influence a range of physiological processes (4, 9, 19). At the cellular level, estrogens promote BDNF expression through the ERα-mediated classic transcriptional pathway in regions such as the hippocampus, amygdala, frontal cortex, dentate gyrus and hypothalamus, subsequently activating MAPKs, PI3K, and PKC pathways (20–22). However, this induction varies across different areas (21), with some studies reporting a decrease in the hippocampus (22, 23). In adipose tissue, the ratio of Bdnf/TrkB (tropomyosin receptor kinase B) expression is higher in female mice than in male mice (24). In addition, estrogens and BDNF may converge to promote the expression of neuropeptide Y (NPY) in hippocampal neurons through the aforementioned pathways (20). BDNF/TrkB signaling activation is essential for ligand-independent ERα activation (25) and is required for the long-term genomic actions of 17β-estradiol on dendritic spine plasticity (26). At the physiological level, their interdependent relationship is supported by evidence showing the fluctuation of BDNF levels during the ovarian cycle in women (19, 27) and in animal models (28). Conversely, reductions in estrogen and BDNF levels have been reported in patients with Parkinson's disease, Alzheimer's disease (AD) (21), postmenopausal women (19), ovariectomized (OVX) mouse models (29, 30), and ER-deficient mouse models (31). Estrogen deficiency is linked to obesity in over 43% of menopausal women (32), characterized by a central reduction of BDNF levels, while plasma BDNF remains unaffected (33, 34). It is also associated with hypertension in 19% of premenopausal women, 44% of perimenopausal women, and 75% of postmenopausal women aged 65 to 74 (35). Additionally, lower plasma BDNF levels are associated with significantly poorer memory performance (36). Although plasma BDNF is believed to influence blood pressure regulation, studies have produced conflicting results: some report elevated BDNF levels in cases of hypertension (1), while others indicate reduced levels (37).

Physiologically, BDNF is recognized as an anti-obesity molecule, while estrogens promote lipolysis and help prevent obesity. Furthermore, BDNF can contribute to increased blood pressure, whereas estrogens aid in maintaining blood pressure within a healthy range. Both BDNF and estrogens play critical roles in the development of hypertension and obesity, particularly in the postmenopausal context. These conditions are significant global public health concerns, affecting approximately 68% of the adults (1, 17, 38). This review examines recent advances in understanding the interaction between BDNF and estrogen in the context of hypertension and obesity. It aims to identify effective therapeutic strategies that leverage BDNF and estrogen, focusing on the timing and selection of appropriate diets or medications.

## 2 BDNF is a key mediator in activity-dependent processes, playing a crucial role from embryogenesis through aging

BDNF is expressed in various tissues, including both CNS and non-CNS organs such as the liver, lungs, kidneys, fat pads, and reproductive tissues. As a target-derived factor, BDNF plays diverse roles in numerous physiological processes, influencing blood pressure, body mass, learning, memory, cognitive development (5), and notably, appetite and metabolic control (39–42). It also has significant contributions to the cardiovascular health (1, 18, 39, 43). BDNF expression begins as early as the 11th to 12th day of embryogenesis in rats and mice, coinciding with prenatal programming, and increases with the onset of neurogenesis and heightened neuronal activity during development (44). Additionally, BDNF expression in target organs and tissues can be enhanced by exposure to various substances, including alcohol (45, 46), cocaine (47–49), exercise (50), high-fat diet (51, 52), low-level of ozone (O_3_) (53), lead (54), cigarette or cannabis smoke (55, 56), and drugs like valproate (57) in rodents or humans. Conversely, prenatal BDNF expression is downregulated by factors such as viral infection (50) or other stressors, including depression and estrogen deficiency (54). Importantly, BDNF plays a critical role in transmitting drug-induced phenotypes to subsequent generations, as observed in women with exposures to alcohol (10, 11), cocaine (12, 13), exercise (14, 15) and high-fat diet (16, 17).

Postnatally, central expression of BDNF significantly increases, influenced by various factors, including estrogens (9), high salt intake (58), angiotensin II or aldosterone (1, 59), exercise (60, 61), intestinal microbial colonization (62), chlorpyrifos (63), cocaine addiction (64), and moderate alcohol consumption (65). This activity-dependent increase in BDNF levels likely results from the stimulation of N-Methyl-D-aspartate ionotropic glutamate receptors (NMDARs), leading to intracellular Ca^2+^ influx. This influx activates Ca^2+^/cAMP-responsive element binding protein (CREB), which binds to the BDNF promoter to initiate transcription (66). The cumulative effect of BDNF is observed with stimuli that lead to persistent and specific changes, particularly in the central nervous system. This heightened sensitivity, shaped by dietary habits and life experiences, enhances environmental adaptation, as seen in the appropriate increase in blood pressure (1, 59, 67). These factors influencing BDNF regulation and their underlying expression mechanisms are summarized in **Table 1**.

**Table 1:** Representative references for prenatal and postnatal stimuli influencing BDNF expression and the underlying mechanisms.

Animal or cell model	Stress factor(s)	Brain region(s)/tissue	Mechanisms	References
Obese mice	Maternal obesity	Fetuses, placenta	miRNA-210↑-BDNF↓(f)/proBDNF (m)	Prince et al., 2016 (68)
Obese rat**s**	Maternal HFD/obesity± resveratrol	Placenta, fetal brain	Restores BDNF, BP↑	Hsu, et al., 2020 (69)
OVX rats	OVX	Brain areas	E2→BDNF↑	Kiss et al., 2012 (70)
Neurons	Estrogen	cerebral cortex and the olfactory bulb	ERE-Like Motif in the BDNF Gene	Sohrabji et al., 1995 (9)
Female rats	Estrogen, stress	hippocampus	Estrogen→BDNF↓	Cavus and Duman 2003 (22)
Maternal Exercise and LP treated dams	Maternal low protein; physical activity	Placenta; dam hypothalamus; hippocampus	Ex→BDNF↑; LP→ BDNF↑	Fragoso et al., 2021 (71)
SD rats	BDNF overexpressed, BP↑	PVN, Astrocytes in the mediobasal hypothalamus	NMDAR↑ and GABAAR↓; IKKβ/NF-κB↓	Thorsdottir et al., 2021 (18); Zhang et al., 2017 (72)
SD rats	10 nM E2	Hippocampal slices	GPER1 activation →BDNF↑, ERα and ERβ independent	Briz V et al., 2015 (73)
BDNF overexpressed	Target overexpressed BDNF	PVN	β1-adrenergic receptor↓; BP↑	Thorsdottir et al., 2019 (74)
SHR Rat	Exercise, calorie restriction (CR)	hippocampus	Ex→BDNF↑; CR→BDNF↑	Kishi et al., 2015 (75)
SHR rats	exercise	BDNF in quadriceps↓, LV, DG and brain areas, endothelial↑	BDNF‐TrkB‐signaling in DG↑ (hippocampus); eNOS↑; SA↑; proBDNF↓	Wang, et al., 2019 (76); Monnier et al., 2017 (77)
SHR and WKY	Hypertension; exercise	Aortic endothelial BDNF	Hypertension→BDNF↓ Exercise→BDNF↑ Exogenous BDNF dialates aortic rings	Prigent-Tessier et al., 2013 (78)
Human	Exercise in the heat	plasma	BDNF↑; 18y↑>32y↑	Roh et al., 2017 (79)
Clinic data	Stroke, exercise, DM, alcohol, smoking	Plasma BDNF	Exercise→BDNF↑; Other factors→BDNF↓	Chaturvedi et al., 2020 (80)
Chronic mild stress mice	Valsartan and stress	hippocampus	Valsartan restores BDNF level	Ping et al., 2014 (81)
Clinic survey	Pregnant women	Placenta BDNF	NC: BDNF differentially in placenta; PE not	Sahay et al., 2020 (82)
Inflammation (cell model)	PGE2	astrocytes	BDNF release↑	Hutchinson et al., 2009 (83)
Inflammation (cell model)	TNF-alpha	astrocytes	BDNF release↑	Giralt et al., 2010 (84)
Inflammation	LPS, 8-ceramide	microglia	BDNF maturation↑; BDNF release↑ via A_A2_R-PKA/PLC	Gomes et al., 2013 (85).
Pain	high-frequency stimulation (HFS; 100 Hz, 10 V)	Microglia in spinal dorsal horn	BDNF release↑	Zhou et al., 2019 (86).
chronic migraine	nitroglycerin	microglia	BDNF release↑via Ras/p38	Long et al., 2020 (87)
Human test, animal model, cultured cells	caffeine, glutamate	CNS, stratum radiatum	antagonism of adenosine and GABAA receptors, IRS, PI3K/Akt	Lao-Peregrín et al., 2017 (88)
Oxidative stress (cell model)	6-hydroxydopamine	astrocytes	BDNF release↑	Datta et al., 2018 (89)

AA2R, Adenosine A2; ACEI, angiotensin converting enzyme inhibitor; Arc, hypothalamic arcuate nucleus; ATRB, angiotensin receptor blocker; CAPS1, Ca^2+^-dependent activator protein for secretion; DG, dentate gyrus; DM, diabetes mellitus; eNOS, endothelial nitric oxide synthase; Ex, Exercise; HS, High salt; IRS, insulin receptor substrate 2; LP, low protein; LPS, lipopolysaccharides; LV, left ventricle; MMSE, mini-mental state examination; NC, normotensive control; PE, preeclampsia; PVN, paraventricular nucleus; RVLM, Rostral Ventrolateral Medulla; SD, Sprague-Dawley rats; SHR, spontaneously hypertensive rats; SNA, sympathetic nerve activity; SON, supraoptic nucleus: WKY, Wistar Kyoto rats

The regulatory effect of estrogens on BDNF is particularly evident in case of estrogen deficiency. Both amenorrheic individuals and postmenopausal women exhibit significantly lower plasma BDNF levels compared to fertile females, and hormone therapy effectively restores BDNF levels in these patients (90). Additionally, the administration of estradiol increases BDNF levels in ovariectomized animals across all ages (70). These findings further underscore the interaction between estrogens and BDNF, particularly in postmenopausal women and animal models.

Enhancing central BDNF levels through the peripheral administration of specific drugs offers a promising strategy for delaying age-related neurodegenerative diseases (91) and ameliorating many of the symptoms discussed above. Alternatively, long-term exercise training enhances brain function and helps prevent neurological disorders by stimulating brain plasticity through the induction of BDNF expression (92, 93). This expression is essential for certain forms of hippocampal-dependent information storage and memory (94). The benefits of exercise training can persist for an extended period, as evidenced by spatial learning and memory tests conducted in both rodents and humans (94, 95).

Therefore, plasma or serum BDNF serves as an endocrine molecule and is proposed as a biomarker for various diseases, including hepatic encephalopathy (96), depression (61, 97), Alzheimer's disease (98), mood disorders (99), schizophrenia (100), neuropsychiatric disorders (101), obesity (102), psoriasis (103), cardiometabolic problems (104), and glaucoma (105), among others. Additionally, BDNF may serve as a useful biomarker for assessing impaired memory and general cognitive function in aging women (106), as well as for prenatal hypertensive anxiety and depression in both rats and post-partum women (107).

However, studies have shown that a chronic reduction of BDNF does not exacerbate the development of neurodegenerative diseases like a Alzheimer's in mouse models (108). Interestingly, BDNF levels in the hippocampus of postmortem brain samples from AD patients are significantly higher compared to age-matched non-demented controls (98). These conflicting data may reflect the complexities of the aging brain, which can be both a consequence and a causative factor to pathological development. Furthermore, the original source of circulating BDNF remains largely unclear (109).

## 3 The role of BDNF and estrogen in body mass regulation

The identification of BDNF as a key gene linked to obesity highlights its crucial role in metabolic regulation (110), affecting both the CNS and peripheral organs (111). This association is particularly evident in individuals with WAGR syndrome (Wilms' tumor, aniridia, genitourinary anomalies, and intellectual disability), where those with heterozygous BDNF deletions exhibit approximately half the serum BDNF levels and a higher incidence of childhood-onset obesity, compared to those with an intact BDNF sequence (112). Additionally, central BDNF knockdown leads to hyperphagia and obesity (39, 113), while the knockout of Trek B in adipocytes reduces HDF-induced obesity in female conditional knockout mice, but this effect is not observed in males (24).

Similarly, global ERα knockout (114) leads to the development of metabolic syndrome characteristics in animal models, including weight gain, increased visceral adiposity, hyperphagia, hyperglycemia, and impaired energy expenditure through the PI3K pathway (115). In contrast to the BDNF’s central effects, estrogens act in the arcuate nucleus (ARC) to suppress food intake via ERα in pro-opiomelanocortin (POMC) neurons and NPY neurons (116, 117). In the ventromedial nucleus of the hypothalamus (VMN), estrogens influence obesity primarily by enhancing energy expenditure, mediated by VMN nitric oxide (NO) and γ-aminobutyric acid (GABA) neurons, involving both ERα and GPER. The role of ERβ, however, varies depending on the experimental model used (114, 118, 119). Additionally, in the nucleus of the NTS, estrogens inhibit food intake by sensitizing satiety signals induced by cholecystokinin (CCK) through the activation of ERα (120). Overall, estrogens contribute to maintaining a healthy lifestyle by promoting balanced nutrition and well-being in both sexes (121–123).

### 3.1 Maternal HFD induces prenatal central BDNF deficiency and offspring obesity

Maternal eating habits play a significant role in influencing offspring health, highlighting the critical role of BDNF in energy balance (124). An optimal fatty acid profile in a mother's diet is essential for the well-being of both mother and fetus. Clinical and experimental evidence suggests that an over-nutritious maternal HFD environment can lead to extensive molecular and cellular changes in the offspring's brain through epigenetic modifications. These changes may include downregulation of BDNF, mutations in the BDNF gene and/or its receptor, and alterations in downstream signaling pathways in the brain, all of which can contribute to neurodevelopmental disorders in the offspring (52). Additionally, maternal HFD impacts the epigenetic programming of appetite and energy homeostasis in the fetus, playing a crucial role in the development of childhood obesity (125). This evidence aligns with the phenotype associated with central BDNF knockdown (Kd) (39, 113), and reduced hypothalamic BDNF expression has been observed in leptin-receptor-deficient db/db obese mice (126).

HFDs induce the expression of neuropeptide Y (NPY) and agouti-related protein (AgRP) in orexigenic neurons, while downregulating pro-opiomelanocortin (POMC) and cocaine- and a mphetamine-regulated transcript (CART) in anorexigenic neurons. These changes occur in various hypothalamic nuclei, including the ventromedial nucleus (VMN), dorsomedial hypothalamus (DMH), lateral hypothalamus (LH), and paraventricular nucleus (PVN) in adults. BDNF and its receptor TrkB are expressed in these regions, with BDNF being most abundant in the VMN under normal dietary conditions. Maribel Rios (126) has elucidated the feeding circuits within these hypothalamic nuclei, demonstrating that HFD-induced changes in these circuits can disrupt appetite regulation and energy balance, potentially leading to obesity.

### 3.2 Hypothalamic BDNF decreases food intake and increases energy expenditure

Postnatal animal models demonstrate that hypothalamic BDNF suppresses food intake by acting on both orexigenic and anorexigenic neurons (39, 113). Consistently, genetically engineered rodents with CNS BDNF knockdown develop hyperphagia and obesity (113, 127, 128). Similarly, individuals with Rett syndrome, characterized by a deficiency in central BDNF, are reported to have a higher risk of obesity (129).

The cellular mechanism involves central BDNF activating the sympathetic nervous system via the Ca^2+^-CREB signaling pathway (1, 59, 130, 131). The cumulative effect of central BDNF activity reduces appetite by increasing the expression of anorexigenic molecules and decreasing the expression of orexigenic molecules in the hypothalamus (39). Additionally, it enhances energy expenditure by boosting sympathetic nerve activity (130), ultimately leading to a reduction in body mass.

### 3.3 Central estrogens decrease appetite, increase energy expenditure and promote weight loss

Estrogen is primarily produced in the ovaries in females, but it is also produced by the adrenal glands and adipose tissue in both males and females (132). Additionally, the CNS can produce estrogens, as it contains all the necessary enzymes for this process. Forebrain-specific knockout of aromatase, the rate-limiting enzyme for neuronal estrogen production, leads to a significant reduction in synaptic density and related functions in mice (133). As adipocyte enlarge, the expression of aromatase in these cells increases, resulting in elevated estrogen levels (16, 121, 134), particularly in postmenopausal women, where this contribution constitutes a substantial portion of endogenous estrogens (134).

Regardless of their sources, estrogens predominantly exert a catabolic effect by interacting with anorexigenic and orexigenic neurons in the hypothalamic arcuate nucleus (ARC). The arcuate nucleus plays a critical role in long-term energy balance, integrating signals from a variety of hormones, including estrogens and leptin (135, 136). Estrogens activate POMC neurons in the ARC, which in turn inhibit NPY/AgRP neurons, leading to reduced food intake (137). Estrogens modulate POMC neuron activity and inhibit AgRP/NPY neuron activity in the ARC through genomic pathways, Gq-coupled membrane ERα (137), and ERα-independent mechanisms (138). They enhance the phosphorylation of protein kinase B, activating a key neuronal signal pathway (139), including protein kinase C, protein kinase A, phosphatidylinositol 3-kinase, and mitogen-activated protein kinase (117, 140, 141). Additionally, estrogens increase POMC neuronal activity and reprogram synaptic plasticity in the arcuate nucleus via a signal transducer and activator of transcription 3 (STAT3)-dependent mechanism, ultimately reducing feeding. Notably, this signaling pathway operates independently of leptin (142). A comprehensive summary of the neuronal circuit and estrogenic signaling pathways can be found in the work of Mahboobifard et al. (141). The ventromedial nucleus of the hypothalamus (VMH) is a key site where both E2 and BDNF act on energy expenditure, primarily receiving projections from AgRP/NPY and CART/POMC neurons in the ARC (143, 144). Estrogen centrally inhibits AMP-activated protein kinase (AMPK) selectively in the VMH through ERα, enhancing sympathetic nervous system-brown adipose tissue (SNS-BAT) signaling and promoting thermogenesis in brown adipose tissue (BAT) (145). This results in increased glucose transport and uptake, aerobic glycolysis, and mitochondrial function, ultimately boosting ATP product, energy expenditure, and weight loss. Moreover, estrogens can also activate the Gq-coupled membrane estrogen receptor (Gq-mER) in NPY/AgRP neurons, which enhances the GABAergic postsynaptic response, however, ERα activation by E2 attenuates this effect. This highlights a functional dichotomy in the central estrogenic regulation of energy homeostasis, contrasting the rapid membrane-initiated signaling via ERα with that of Gq-mER in CNS neurons. (116). Additionally, estradiol administration has been shown to attenuate skeletal sympathetic nerve activity responses to exercise in postmenopausal women (146), indicating suggesting that estrogen may regulate sympathetic activity in a specific and conditional manner.

In addition, estrogens inhibit food intake by enhancing cholecystokinin (CCK)-induced satiety, which involves increased activity of NTS neurons through binding to ERα. This interaction regulates target gene expression, including the upregulation of c-fos (147–149) and postsynaptic density 95 (PSD-95) (139). Additionally, estrogens amplify other appetite-reducing signals, such as apolipoprotein A-IV (apo A-IV) (150) and glucagon-like peptide 1 (GLP-1) (151) within the NTS to further reduce food intake. Furthermore, BDNF/TrkB signaling in the NTS serves as a downstream mediator of estrogen's effects on energy intake, specific knockdown of BDNF in the NTS diminishes the feeding response to estrogens (152).

### 3.4 The role of adipocytic BDNF in the peripheral regulation of fat mass

The peripheral effects of BDNF on cellular functions and the associated signaling pathways related to metabolism have been demonstrated (24, 153) and thoroughly reviewed by Iu and Chan (24, 111). In contrast to the lower BDNF levels in the CNS observed in HFD-induced obese mice, these mice exhibit higher levels in inguinal white adipocyte tissue (iWAT) and epididymal white adipose tissue (eWAT) compared to controls, with this increase being macrophage-dependent (154). Adipocyte-specific TrkB knockout mice show resistance to HFD-induced obesity in females (24). Conversely, fat pads in systemic BDNF knockdown mice still respond to HFD stimulation by secreting more leptin than controls (155). Moreover, BDNF knockout leads to obesity (39), indicating that adipocytic BDNF is essential for the central-peripheral BDNF regulatory loop, which integrates central appetite signals and adipokine levels (155). Without adipocytic BDNF, the obese phenotype resulting from central BDNF deficiency cannot manifest, thus, the presence of adipocyte BDNF is necessary for expressing obesity due to central BDNF deficiency.

### 3.5 Peripheral effects of estrogen on body mass

Fat pads serve as the primary extragonadal sites for estrogen production, acting locally in a paracrine fashion or being released into circulation, particularly in postmenopausal women, men, obese individuals, and other cases (121). Peripherally, estrogens exert various metabolic effects, including increasing mtDNA polymerase Polg1 levels and mitochondrial content in WAT through ERα, thereby enhancing energy expenditure (156). They also improve insulin sensitivity by promoting energy sensing and glucose uptake via the Akt-AMP-activated protein kinase (AMPK) pathway in skeletal muscles (157). Additionally, estrogens reduce the expression of hepatic lipogenic genes, such as FASN, acetyl CoA carboxylase (ACC), and stearoyl CoA desaturase 1(SCD-1), through the STAT3 signaling pathway in the liver (158, 159). Furthermore, estrogens strongly inhibit key adipogenic genes, such as PPARγ, CBP, and adipsin, as well as leptin production, while increasing hormone-sensitive lipase expression and reducing adipocyte size (15, 160). Notably, estrogens significantly influence body fat distribution, favoring the accumulation of metabolically healthy subcutaneous fat in females while promoting visceral fat accumulation in males or OVX females (15, 15, 161, 162). The actions of estrogens in adipose tissue also extend to influence adipocyte differentiation (163) and reducing inflammation (164). Through ERα activation, estrogens provide protection against adiposity, insulin resistance, and type II diabetes while simultaneously increasing energy expenditure (165, 166). In this context, ERβ has a counteractive effect against ERα (166).

Similar to central estrogen, peripheral estrogens can also correct the abnormal appetite and metabolism resulting from central BDNF deficiency. This correction involves the transcriptional regulation of metabolic enzymes, including the downregulation of fatty acid synthase (167) and the upregulation of hormone-sensitive lipase (168). In summary, estrogens integrate brain and body metabolism, encompassing the effects of BDNF on metabolic processes, enabling the peripheral metabolic state to reflect the brain's bioenergetic status (141).

### 3.6 The collaborative effect of estrogen and BDNF on energy expenditure and body weight

In animal models, estrous rats undergoing sham surgery and ovariectomized rats cyclically treated with estradiol exhibit increased sensitivity to lower doses of centrally administered BDNF, leading to reduced food intake compared to male rats and oil-treated ovariectomized rats (131). This finding suggests a cooperative effect between estrogen and BDNF in regulating food intake. Although a tri-molecular cascade model—estrogen-BDNF-NPY/AgRP—has been established in the hippocampus and dentate gyrus, its direct evidence in the hypothalamus, particularly in the arcuate nucleus (ARC) and ventromedial hypothalamus (VMH), remains limited (20). Given that the estrogenic effect on BDNF expression is highly location-specific, research is needed to elucidate their relationship (21, 22, 73). The following paragraphs will focus on detailing their interaction, supported by direct evidence from the nuclei in the hypothalamus and brainstem, including the ARC, ventromedial hypothalamus (VMH), and nucleus tractus solitarius (NTS).

The ARC is a key site for the actions of steroids, BDNF, and leptin action, mediating leptin's effects through the antagonistic activity of POMC and AgRP/NPY neurons (135, 169, 170). These microcircuits play crucial roles in energy homeostasis: AgRP/NPY neurons signal hunger and stimulates food intake, while POMC neurons signal satiety and reduces food intake (171, 172). leptin acts as a monitor of energy balance within the system (173). Estrogens activate POMC neurons and inhibit AgRP/NPY neuron activity through ERα-dependent genomic and membrane-coupled pathways (137), as well as ERE-independent signaling (117, 138, 140). In contrast, the ARC expresses little to no TrkB in neurons that produce cocaine– and amphetamine–regulated transcript (CART) or NPY, suggesting that BDNF likely serves as a downstream effector of melanocortin-4 receptor (MC4R) signaling to decrease the NPY/AgRP neuron activity (173). MC4R is activated by α-melanocyte-stimulating hormone (α-MSH), a posttranslational product of POMC, which increases BDNF expression through the classic cAMP-Protein kinase A-cAMP responsive element binding protein (CREB) pathway and the ERK-ribosomal p90 S6 kinase (RSK)-cFos pathway in the rat hypothalamus (174). These evidence supports a model of estrogen-BDNF interplay in the ARC, independent of genomic estrogen effects on BDNF expression (9). This model posits that estrogen, acting in concert with POMC/ α-MSH, MC4R, BDNF, and NPY, modulates food intake regulation (173). Additionally, rapid, non-genomic estrogen signaling and acute BDNF signaling have been shown to promote dendritic spine formation and stabilization, supporting synapse and circuit plasticity while synergistically inhibiting appetite (26).

The VMH is crucial for regulating satiety, with BDNF primarily expressed there through its promoters II (175).The VMH-specific expression of BDNF and Trek B is essential for the suppression of appetite (175). Mutation in BDNF promoters II or Trek B deficiency in the VMH produce phenotypes similar those observed in leptin-deficient (Ob/Ob) mice (170), establishing BDNF as an integral component of central mechanisms mediating satiety (113). BDNF neurons in the VMH are activated by ARC POMC neurons (176, 177), which are also activated by estrogens in the ARC. This activation occurs through the inhibition of the small conductance of the calcium-activated potassium (SK) channel (178), as well as through ERα dependent signaling and c-Fos mediating cascades (116, 117). Moreover, both estrogen and BDNF work together to maintain mGluR5 function, regulating the firing rate, intrinsic excitability, and excitatory and inhibitory transmission in VMH neurons, thereby facilitating glycemic control and lipid metabolism (179). Their cooperation may dependent on ERα (180) and GPER1 signaling (181). However, VMH BDNF primarily exerts its anorexigenic effects through Trek B signaling, interacting indirectly with the leptin pathway (182), while estrogens mainly enhance sympathetically driven thermogenesis (118, 180).

In the NTS, estrogens increase BDNF expression by binding to ERα, but not ERβ, thereby initiating estrogen's genomic effect (20). Similarly to the role of BDNF in the ARC, BDNF/TrkB acts downstream of estrogen-ERα signaling; knocking down BDNF or administering a selective TrkB antagonist in the NTS prevents the anorexic effect of estrogen (152). This suggests that estrogens enhance BDNF's satiating potency, involving CCK-CCKR1 in leptin receptor-positive neurons in the NTS (183, 184). Estrogens also increase the expression of apo A-IV, a satiation factor from the gut and brain, through cytosolic ERα (150). They interact with apo A-IV via the cell membrane-bound ERα-PI3K/Akt signaling pathway to reduce food intake (26, 139, 185), while ERβ appears to have no effect on these pathways (186). Currently, there is no evidence indicating that BDNF is involved in the apo A-IV-mERα-PI3K/Akt pathway.

Non-CNS BDNF may increase in response to the loss of central BDNF induced by HFD (154). Similarly, extragonadal estrogen levels rise in enlarged fat pads due to increased 11β-HSD1 activity, which is triggered by central BDNF deficiency (39, 128, 187) and also observed in postmenopausal women (121). This suggests that peripheral estrogen and BDNF may compensate for the lack of central BDNF; however, this compensation may lead to a high equilibrium body weight (188). Notably, leptin exhibits antidepressant-like effects (189). Peripheral estrogen exerts autocrine and paracrine effects that contribute to increased body mass, alongside elevated BDNF and leptin levels, in the context of central BDNF deficiency— an occurrence referred to as "obesity protecting obesity" (132, 188). In contrast, under normal BDNF levels, estrogen finely tunes lipogenesis in various tissues, supporting a healthy metabolism (165).

### 3.7 Leptin: a key intersection of BDNF and estrogens

Under most circumstances, body energy levels are primarily sensed through circulating leptin levels (190). The presence of ER, leptin receptor, and BDNF/TrkB in POMC neurons within the ARC indicates that leptin significantly influences the interplay between BDNF and estrogens in regulating energy homeostasis (191, 192). Hypothalamic BDNF downregulates leptin production in adipocytes via sympathoneural β-adrenergic signaling (193). In contrast, central BDNF knockdown leads to obesity and elevated leptin expression in adipocyte (39). This increase in fat raises both estrogen levels (121) and adipokine levels, including leptin (128, 194). In normal cycling women, leptin levels positively and strongly correlate with estrogen levels, and increases further with larger fat depots (195).

Leptin reduces appetite by binding to the leptin receptor, particularly ObRb, in the arcuate (ARC), VMH, and DMH nuclei of the hypothalamus, triggering signal pathways like STAT, PI3K, and ERK (196). Centrally, leptin augments POMC neuron activity via BDNF-expressing neurons in the hypothalamic ARC, a process known as the leptin–BDNF pathway, which alters the sympathetic architecture of adipose tissue through a top (ARC)-down (PVN) neural mechanism (141, 170). Estrogens sensitize the anorexigenic effect of leptin by increasing the expression of the leptin receptor through genomic pathways and by potentiating leptin-induced pSTAT3 activation in the hypothalamus (197). Furthermore, leptin promotes local estrogen production in adipocytes by upregulating the aromatase expression and activity via STAT3 and ERK signaling pathways (198).

Estrogen deficiency, seen in ovariectomized (OVX) mice (70) and postmenopausal women (141), along with central BDNF knockdown (39), leads to increased fat accumulation and elevated leptin level. Adipocyte-specific deletion of BDNF/TrkB results in resistance to HFD-induced obesity, particularly in females (24),indicating that adipocytic BDNF is essential for the adipocytic response to central BDNF signaling and the production of adipocytokines, including leptin. Additionally, activation of mERα/mERβ can reduce body weight gain and fat accumulation in ovariectomized (199) and leptin-deficient obese mice through the PI3K pathway (200), suggesting that mER signaling can regulate energy balance independently of leptin signaling. Thus, part of the protective effects of estrogen and BDNF on energy homeostasis involves leptin (201), which may support the effects of their deficiency. It is possible that estrogen facilitates, or mimics some leptin actions (190), indicating that their interplay in regulating energy homeostasis is complex and warrants further combined studies rather than isolated examinations.

**Figure 1:**
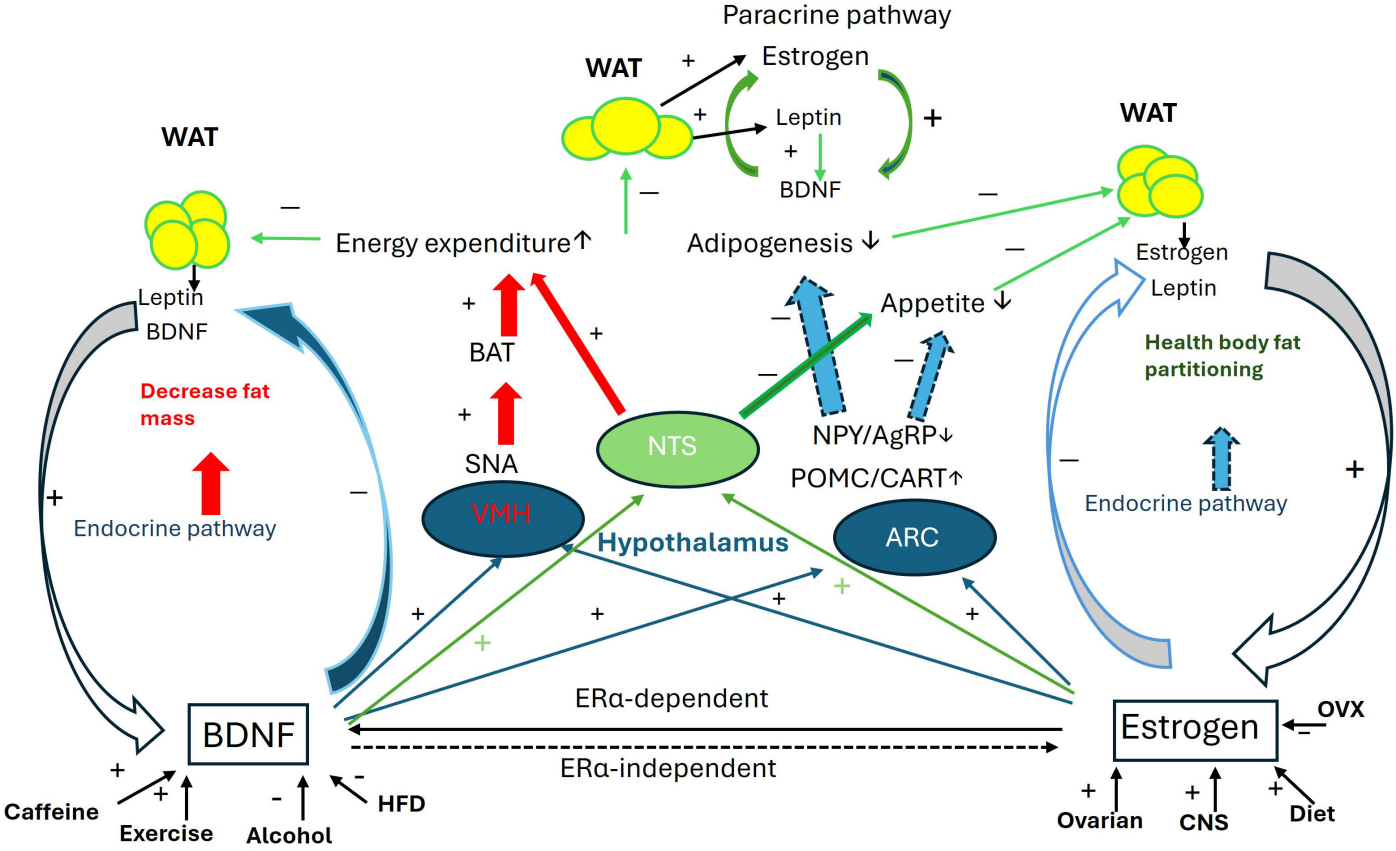
Collaborative interaction between BDNF and estrogen in the regulation of body mass. Schematic of the collaborative interaction between BDNF and estrogen through 1. Mutual activity enhancement: estrogen directly upregulates BDNF expression via the ERα-dependent genomic pathway, while BDNF is essential for estrogen’s effects through ERα-independent non-genomic pathways. 2. Regulation of VMH function: Both BDNF and estrogen enhance energy expenditure through the VMH-SNA-BAT pathway. 3. Regulation of NTS function: With the modulation of leptin released from fat pads, both BDNF and estrogen potentiate anorexic effects and suppress orexic effects to increase energy expenditure and reduce appetite. 4. Regulation of ARC function: BDNF and estrogen collaborate in the ARC to upregulate POMC and CART expression while downregulating NPY and AgRP expression, thus regulating appetite. Additionally, estrogen and BDNF mutually enhance their expression in fat pads. For more details, please refer to the relevant sections in the text. AgRP, agouti-related peptide; ARC, arcuate nucleus; BAT, brown adipocyte tissue; CART, cocaine- and amphetamine-regulated transcript; CNS, central nerve system; HFD, high fat diet; NPY, neuropeptide Y; NTS, Nucleus Solitarius; OVX, ovariectomy; POMC, Pro-opiomelanocortin; SNA, sympathetic nerve activity; VMH, ventromedial nucleus; WAT, white adipocyte tissue. + increase; − decrease.

The collaborative influence of BDNF and estrogen on body mass regulation is illustrated in **Figure 1**.

### 3.8 Evidence for the interplay Between BDNF and estrogen in the liver and skeleton muscle

Peripheral organs such as muscles and the liver play vital roles in the central-peripheral circulation alongside fat pads. The functions of hepatic and muscle BDNF are extensively reviewed by Lu and Chan (111). Muscle-specific BDNF is essential for the regulatory loop that maintains energy balance; muscle-specific BDNF knockout (MBKO) mice exhibit impaired mitofission and mitophagy, leading to exacerbated body weight gain, reduced energy expenditure, and poor metabolic flexibility (202). Similarly, BDNF deficiency in the liver impairs metabolic regulation, resulting in hepatic steatosis and obesity (127). The deficiencies of BDNF in both liver and skeletal muscles contribute to obesity, highlighting a complex interaction among these organs that extends beyond the scope of this review.

## 4 The interaction between BDNF and estrogen in the development of hypertension

### 4.1 BDNF enhances response to recurrent, sustained, or new stressors following blood pressure stimuli

Blood pressure is directly regulated by the renin-angiotensin system (RAS) and its counteracting system. The activity of RAS is influenced by a diverse of plasma signals released from organs or tissues, such as the CNS, kidneys, lungs, liver, and adipose tissue, all of which are enriched with BDNF (1, 203). Changes in the levels of individual RAS components, such as angiotensinogen (AGT) or renin, may not directly correlate with blood pressure (unpublished data). However, the CNS effectively encodes these signals by modulating central BDNF expression, which facilitates neuroplasticity (1). The reconfigured neural network allows the brain to adaptively respond to recurring, sustained, or novel stressors.

The role of BDNF in responding to hypertensive stimuli during prenatal embryogenesis and postnatal adaptation has been thoroughly reviewed by Manti et al. (204) and Johnson et al (1). **Table 2** summarizes key animal models and clinical studies that highlight the causative factors of brain’s hypertensive response through BDNF-related pathways.

**Table 2:** Representative references on the adaptive expression of BDNF in response to hypertensive stimuli including high salt, Ang II, and high fat.

Animal or cell model	Stress factor(s)	Brain region (s)	Mechanisms	References
Subpressor doses priming (mouse model)	ANG II; aldosterone; high salt	PVN and RVLM	BDNF↑; p38 MAPK, and cAMP-CREB	Clayton SC, et al., 2014 (59)
High salt mouse model	Na^+^, Cl^-^	VP Neurons	BDNF↑-TrkB-KCC2↓-VP MNCs	Choe et al., 2015 (58); Prager-Khoutorsky, et al., 2017 (205)
High salt mouse model	amlodipine and irbesartan	cerebral vessels	BDNF↑-stroke↓	Hasegawa et al., 2016 (206)
SD rats	BDNF overexpressed, BP↑	PVN, Astrocytes in the mediobasal hypothalamus	NMDAR↑ and GABAAR↓; IKKβ/NF-κB↓	Thorsdottir et al., 2021 (18); Zhang et al., 2017 (72).
BDNF SON Kd	High salt (HS)	SON	HS-SON BDNF↑-VP↑, but not MAP;	Balapattabi et al., 2018 (207)
Conditional CNS knockout	Ang II, nervous system BDNF (+/-)	CNS	BDNF↓-RAS↓-BP↓; BM↑;resistant to AngII-induced HT	Zhang et al., 2019 (39)
Complete BDNF knockout	Bdnf+/− rats	BM↑, hepatic ALAT↓, liver regeneration &steatosis↑, IL-6↑	BDNF-liver regeneration↓, BM↓ (similar to CNS BDNF Kd)	Grezlak et al., 2023 (127)
High salt +NPY Arc targeting overexpression	NPY+/-HS	ARC	HS- NPY↓-BDNF↑-VP↑-MAP	Zhang et al., 2022 (208)
Central Ang II-induced mice	Ang II	Central BDNF and BP	BDNF↑-SNA↑-BP↑	Becker et al., 2017 (209)
Clinic survey	trans fat intake	Plasma BDNF	Low BDNF correlates with hypertension	Harlyjoy et al., 2023 (37)
Clinic survey (dimorphism)	Obesity, age	platelet and plasma BDNF	BDNF↓ with BW, Age; platelet BDNF Man>Woman	Lommatzsch et al., 2005 (210)
Clinical trial on antihypertensive medication, MMSE	ACEI, ATRB	Plasma BDNF; SBP	SBP↓, plasma BDNF (P = 0.09) (3-month treatment)	Demir et al., 2016 (211)
AD (cell culture)	Amyloid-β	hippocampal neurons	Impaired BDNF transportation	Seifert et al., 2016 (212)
AD (cell culture)	Amyloid-β 42	neuroblastoma cell line	BDNF release↑	Merlo et al., 2018 (213)

ACEI, angiotensin converting enzyme inhibitor; AD, Alzheimer's disease; Arc, hypothalamic arcuate nucleus; ATRB, angiotensin receptor blocker; BM, body mass; BP, blood pressure; CNS, central nervous system; HS, high salt; HT, hypertension; Kd, knock down; MMSE, mini-mental state examination; RAS, renin-angiotensin-system; HS, High salt; PVN, paraventricular nucleus; RVLM, Rostral Ventrolateral Medulla; SA, sympathetic activity; SD, Sprague-Dawley rats; SON, supraoptic nucleus; VP, Vasopressin.

### 4.2 Slow pressor-induced hypertension: animal models demonstrating the gradual cumulation of BDNF effects on enhanced hypertensive responses

Pressor agents, such as high salt/AngII, are commonly used to study hypertension development in animal models. These agents increase BDNF levels in the hypothalamic paraventricular nucleus (PVN), enhancing neuronal activity in this region. This heightened activity stimulates the release of vasopressin (VP), activates downstream signaling pathways that raise BP (59, 207), and increases expression of RAS components (1, 59, 207).

In models employing low-dose salt and AngII induction, a method known as subpressor priming, animals display a progressively heightened hypertensive response (1, 59). This priming, achieved with low dose of salt (59), Ang II (214) or aldosterone (215), sensitizes animals to Ang II-induced hypertension by increasing BDNF levels in the PVN. This suggests that BDNF serves as a critical hub for multiple pathways, enhancing RAS sensitivity and exacerbating the development of hypertension. Johnson et al. (1) illustrate the central circuitry of BDNF, detailing its signaling pathways and physiological effects, including its impact on sympathetic tone and RAS component expression.

However, the roles of BDNF in RAS sensitization by other factors, such as inflammatory agents (216) and predator scent stress (217), remains an enigma. The observed decline in plasma and serum BDNF levels with age in humans (218), along with rising blood pressure, suggests the involvement of additional factors, including sex hormones (219). Notably, microinjection of 1 nmol/L BDNF into the subfornical organ (SFO) of anesthetized rats has been shown to decrease blood pressure (220), indicating that this effect may be context-dependent rather than solely attributable to BDNF.

### 4.3 Central maintenance of estrogen in regulating blood pressure

The impact of estrogens on hypertension is evident in the observed sex dimorphism, where adult men generally exhibit higher blood pressure. This distinction is further illuminated by examining blood pressure changes in women from adolescence through puberty and into postmenopause, highlighting the role of estrogen. Additionally, fluctuations in blood pressure throughout the menstrual cycle underscore estrogen's regulatory influence (219).

Estrogenic signals are required for the baseline expression of certain RAS components, such as renin (10, 221), angiotensin-converting enzyme 2 (ACE2) (222) and angiotensinogen (12, 221). Furthermore, endogenous estrogens help sustain normal blood pressure in premenopausal women, who typically have lower blood pressure compared to age-matched men (223). One mechanism for this effect is the vasodilation induced by nitric oxide (NO) and hydrogen sulfide (H2S), both produced by estrogens through ERα, ERβ and GPER-dependent pathways (223, 224). This evidence suggests that estrogens are essential for maintaining blood pressure, rather than merely reducing it.

Unlike BDNF, which potentiates the blood pressure response, estrogens act centrally to counteract these stimuli, contributing to stable blood pressure regulation. This stability is partly due to their transcriptional effects, which inhibit RAS components in an ERα-dependent manner (225). For example, low levels of RAS components are observed in female mice compared to their male counterparts (219) and OVX females (203). This subtle difference likely stems from prenatal fetal programming, and is further amplified by estrogen's effects during puberty, leading to the observed sexual dimorphism in blood pressure between males and females (219). This dimorphism tends to diminish with menopause and aging (219, 226).

Estrogen reduces blood pressure centrally by inhibiting RAS components in the subfornical organ (SFO) and other areas of the lamina terminalis (LT), which are vital for long-term blood pressure and hydroelectrolyte balance in the brain (227). Specifically, estrogens reduce the expression of central RAS components, such as the AT1 receptor and ACE1, in the LT (227). Conversely, central knockdown of ERα negates the protective effect on Ang II-induced hypertension, resulting in a significant increase in AT1, ACE1 and renin, along with a decrease in angiotensinogen (225). Additionally, maternal hypertension sensitizes ovariectomized rats to Ang II-induced hypertension in a sex-specific manner, linked to elevated RAS components in the LT and paraventricular nucleus (PVN). Administration of estradiol through the SFO can partially reverse this prenatal sensitization (228). Notably, BDNF knockdown in the SFO also decreases blood pressure by downregulating RAS components (39), indicating that BDNF and estrogen may antagonize each other in the regulation of blood pressure through their impact on RAS components.

In addition to regulating central RAS components, estrogens play a crucial role in modulating neuronal activity in the rostral ventrolateral medulla (RVLM), a key regulatory center for heart rate, blood vessel constriction, and blood pressure. In the Goldblatt two-kidney one-clip (2K-1C) male rat model of renovascular hypertension, microinjection of 17β-estradiol into the RVLM significantly reduced mean arterial pressure and renal sympathetic nerve activity in control rats compared to experimental rats. This effect is primarily mediated by ERα rather than ERβ (229). Furthermore, GPER may also influence blood pressure regulation, as microinjection of the G protein-coupled estrogen receptor (GPER) agonist G-1 into the RVLM resulted in a marked increase in mean arterial pressure and renal sympathetic nerve activity in experimental rats (229). These findings suggest a counteractive relationship between the genomic and non-genomic estrogen effects in the RVLM, mediated through ERα and GPER (229).

In addition to modulating RAS and sympathetic nerve activity, estrogens play a role in regulating various factors related to hypertension, including vasodilation and fluid balance, allowing for adaptation to environmental changes. For a more comprehensive discussion, please refer to the review by Ashraf and Vongpatanasin (230).

### 4.4 Comparison of BDNF and estrogen in the regulation of RAS activity

Serum BDNF levels influence blood flow and are linked to angiogenesis through TrkB signaling (231). However, it is unclear whether elevated BDNF directly causes increased blood pressure or if changes in blood pressure influence BDNF levels. For example, exogenous BDNF induces vasodilation in aortic rings, while hypertension suppresses BDNF expression in aortic endothelial cells (78).

Estrogens regulate blood pressure by modulating RAS activity through both genomic and non-genomic effects. In contrast to BDNF, which generally increases most RAS components in both the CNS and peripheral organs, except for renin in the kidney (39), estrogens selectively upregulate angiotensinogen levels while downregulating renin levels, angiotensin-converting enzyme (ACE1) activity, AT1 receptor density, and aldosterone production. Consequently, estrogens reduce RAS activity by downregulating most of the RAS components (232). Notably, the transcriptional effects of estrogen on RAS components in peripheral organs are tissue specific. For example, estrogens rapidly and significantly induce angiotensinogen expression in the liver but not in the cardiac atria (221). Additionally, estrogens potentiate vasodilation through eNOS pathway, and attenuate vasoconstriction via GPER signal (233). These patterns suggest that estrogen finely and cooperatively regulates blood pressure by integrating signals from the central nervous system to the peripheral tissues, much like how it shapes fat distribution for a healthy physique.

Overall, central BDNF amplifies the response to hypertensive stimuli by enhancing neuronal plasticity and increasing sympathetic nerve activity. In contrast, estrogens play a key role in establishing the blood pressure setpoint as part of metabolic homeostasis (132) and counteract BDNF-induced deviations through transcriptional regulation of RAS components and by reducing sympathetic nerve activity. The outcome of their interaction can vary significantly depending on factors such as the specific reagents used, cell types involved, and life stages of individuals, such as premenopause, perimenopause and postmenopause.

### 4.5 Leptin enhances the sensitivity of central RAS activity

Estrogen increases the synthesis and secretion of leptin from adipocytes through ERα signaling, but not ERβ (13). Leptin, in turn, sensitizes the body to hypertensive stimuli by enhancing central RAS activity and promoting the release of proinflammatory cytokines (234), while also innervating BDNF neurons in the paraventricular nucleus of the hypothalamus (170). Thus, instead of acting as a collaborative factor with estrogen in energy homeostasis, leptin functions as a counterpart for estrogens in blood pressure regulation. However, leptin resistance is also reported to contribute to hypertension in Bardet-Biedl syndrome mouse models (235), suggesting that obesity paradox may be at play (236), or leptin's effects can vary depending on different physiological backgrounds.

**Figure 2:**
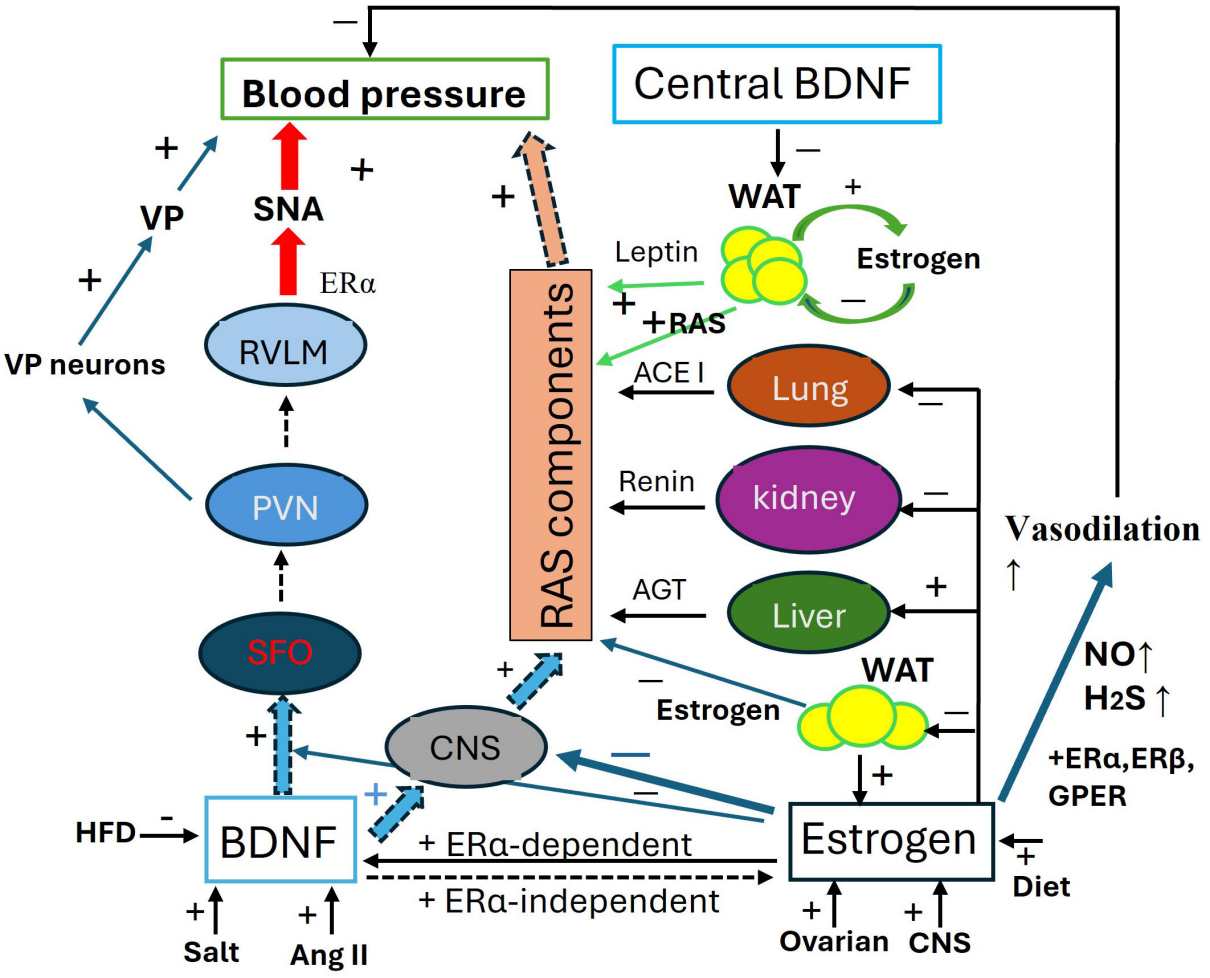
Antagonistic interaction between BDNF and estrogen in the regulation of blood pressure. Schematic of the antagonistic interaction between BDNF and estrogen in the regulation of blood pressure (BP). BDNF increases blood pressure through 1. SFO-PNN-VP Pathway: by activating this pathway, BDNF increases VP release to elevate BP; 2. SFO-PVN-RVLM Pathway, via this pathway, BDNF enhances SNA to increase BP; 3. RAS component expression, BDNF systemically upregulates the RAS components to elevate BP. The expression of BDNF is activity-dependent and influenced by factors such as high salt intake, Ang II, and a high-fat diet. Estrogens maintain baseline blood pressure by transcriptionally regulating RAS components in the liver, kidneys, lungs, CNS, and fat pads. Additionally, Estrogens enhance the generation of H2S and NO for vasodilation through ERα, ERβ, and mGluRs pathways. Estrogens play an integrative role across various organs and tissues, fine-tuning blood pressure regulation, including modulating the effects of BDNF. ACE1, angiotensin converting enzyme 1; AGT, angiotensinogen; AngII, angiotensin II; CNS, central nerve system; ERα/β, estrogen receptor α/β; GPER, G-protein coupled ER; HFD, high fat diet; H2S, hydrogen sulfide; NO, nitric oxide; PVN, paraventricular nucleus; RAS, renin-angiotensin-system; RVLM, Rostral Ventrolateral Medulla; SAN, sympathetic activity; VP, vasopressin.

These pathways (**Figure 2**) illustrate the communication between peripheral fat pads and the hypothalamus, clarifying the roles of both central and peripheral BDNF in the relationship between obesity and hypertension. This model may also account for the sensitization of angiotensin II-induced hypertension in adult offspring that were primed by maternal high-fat diet (237).

The interaction between BDNF and estrogen, along with the role of leptin, is illustrated in **Figure 2**.

### 4.6 Evidence of BDNF and estrogen interaction in body fluid balance

The amount of body fluids fluctuates during normal reproductive cycles, in sync with varying levels of ovarian hormones. Estrogen treatment enhances fluid retention by lowering the threshold for arginine vasopressin (AVP) release and increasing plasma renin activity (238). Studies have demonstrated that water deprivation for 24 hours, 2 days, and 4 days, as well as salt loading for 7 days, result in a significant increase in BDNF gene transcripts in the SFO in rats (239), suggesting the BDNF’s involvement in the regulation of body fluids. However, there is limited direct information available on the specific interaction between BDNF and estrogen in this context.

## 5 Postmenopausal syndrome, a clinic model of estrogen and BDNF deficiency

Estrogen deficiency is a key factor in the onset of menopause. Postmenopausal women experience a decline in plasma estrogen levels, leading to menopausal symptoms, including metabolic changes (110). Both postmenopausal women and amenorrheic individuals exhibit significantly lower plasma BDNF levels compared to fertile females (19, 240), underscoring the complex interplay between BDNF and estrogen highlighted in this review.

Postmenopausal women often experience weight gain, particularly in the form of visceral obesity (162, 241). The weight gain and the accumulation of abdominal fat are likely due to estrogen deficiency, as hormone replacement therapy (HRT) can alleviate these symptoms in postmenopausal women (242, 243). Estrogen deficiency results in a reduction in estrogen-dependent BDNF (9) and ERα (11) expression in target tissues, thereby further weakening estrogenic signals. Ultimately, estrogen deficiency results in a loss of fine-tuned fat accumulation, leaving postmenopausal women with more visceral fat, which becomes the primary source of leptin, adipokine (162, 194, 243), RAS components (244, 245) and even estrogen (134) in postmenopausal women.

Increased visceral fat mass leads to higher synthesis of leptin, which upregulates BDNF in hypothalamic neurons (182). Leptin stimulates lipolysis while inhibiting lipogenesis (246) and enhances thermogenesis in BAT (246). Additionally, it restores sympathetic innervation of WAT (194), acting as a substitute for estrogen by suppressing appetite, increasing energy expenditure and reducing body weight and adiposity. Moreover, leptin modulates the neuroendocrine axes, autonomic nervous system, neural plasticity, and memory, thereby partially replicating the effects of estrogen and BDNF (194).

**Figure 3:**
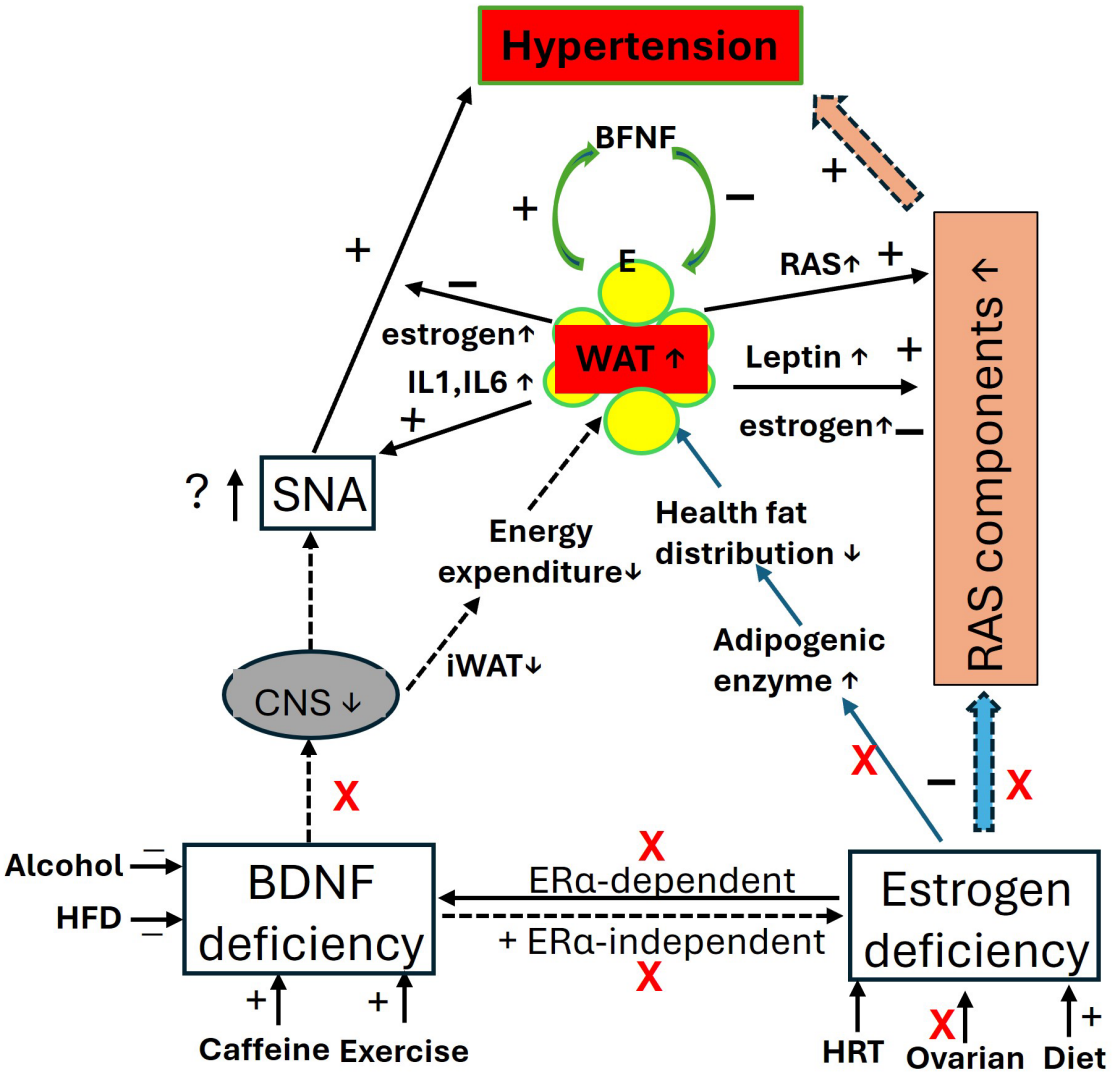
The impact of BDNF and estrogen deficiency in postmenopausal women: focus on blood pressure and obesity. In the context of BDNF and estrogen deficiency, the central effects of BDNF and estrogen, along with their regulation of RAS activity and mutual interactions, are diminished, leading to elevated blood pressure (hypertension) and increased fat mass (obesity). Visceral fat produces more estrogens through the upregulation of estrogen-converting enzymes 17β-hydroxysteroid dehydrogenase (HSD17B7) (134), and synthesizes more leptin with adiposity, which may help inhibit food intake and regulate blood pressure. However, fat pads release proinflammatory agents, contributing to immune and metabolic disorders. iWAT, beige adipose tissue; CNS, central nerve system; E, estrogen; ERα, estrogen receptor α; HFD, high fat diet; HRT, hormone replace treatment; IL, interleukins; RAS, renin-angiotensin-system; RVLM, Rostral Ventrolateral Medulla; SNA, sympathetic activity.

However, alongside aging factors, the elevation of leptin increases sympathetic nerve activity (247) and RAS activity in postmenopausal women and OVX animal models (234, 248), contributing to postmenopausal hypertension. Additionally, elevated levels of adipokines such as IL-1 and IL-6 from enlarged visceral fat pads may promote vascular inflammation, endothelial dysfunction, and increased vascular resistance, further exacerbating hypertension (194, 249, 250). These factors can also trigger the immune system, leaving postmenopausal women vulnerable to chronic inflammatory syndromes (85, 194). These processes are illustrated in **Figure 3**.

Given that central BDNF knockdown leads to resistance to Ang II-induced hypertension (39), while central ERα knockdown results in heightened sensitivity to Ang II-induced hypertension (225), BDNF and estrogen function as physiological antagonists. Specifically, central BDNF contributes to an elevated blood pressure in response to environmental stimuli (1, 67), whereas estrogen’s central effect is to maintain blood pressure within the set point range (251). Reduced signaling from both BDNF and estrogen can impair an individuals' ability to regulate blood pressure in response to environmental changes, potentially leading to hypertension, especially in postmenopausal women.

Although the role of hormone replacement therapy (HRT) with estrogen or/and progesterone remains debated, it is widely used in clinical practice (176). Additional strategies, such as phytoestrogens, combined estrogen and progesterone treatments, and non-hormonal options, have also been proposed (252). These approaches boost plasma estrogen and BDNF levels (19, 240) provided the individual is suitable for such treatments. For safety reasons, lifestyle modifications like regular exercise (79, 80), coffee consumption (88) and a healthy diet, known to increase plasma BDNF and estrogen levels, are highly recommended. Conversely, high-fat diets (253), smoking, and alcohol, which are known to decrease BDNF levels (80), should be approached with caution as they may exacerbate postmenopausal syndromes.
